# Development of the Salford Rheumatoid Arthritis Foot Evaluation Instrument (SAFE)

**DOI:** 10.1186/1757-1146-6-S1-O34

**Published:** 2013-05-31

**Authors:** Steven Walmsley, Anita Williams, Mike Ravey, Andrea Graham

**Affiliations:** 1 School of Science and Health, University of Western Sydney City, Sydney, 2560, Australia; 2 Centre for Sport, Health and Rehabilitation Sciences, University of Salford, Salford, Greater Manchester, M5 4WT, UK

## Background

For clinicians involved in managing the foot with rheumatoid arthritis (RA), there is a dearth of patient-reported outcome measures (PROs) available with only the Leeds Foot Impact Scale (LFIS). The LFIS currently lacks evidence for important measurement properties, does not permit a patient-specific assessment strategy and was designed using a restrictive scaling strategy. Therefore, the aim of this research was to develop a new PRO, the Salford Rheumatoid Arthritis Foot Evaluation Instrument (SAFE), working closely with clinicians and patients, to create an instrument with multiple assessment strategies (fixed and patient-specific) and rigorous measurement properties.

## Methods

Development of the SAFE was divided into 4 stages: conceptual basis and content development, clinimetric instrument development, instrument pre-testing and demonstration of instrument measurement properties, including construct validity and temporal stability of the fixed scale.

## Results

A total of 123 items were initially generated for the SAFE, with 25 of them clinimetrically selected for the fixed scale and 80 items initially included in the patient-specific scale. The pretesting strategy proved effective for improving and refining the SAFE, with the final draft consisting of 19 items in the fixed and 42 items in the patient-specific scale. The fixed scale has strong evidence for validity and temporal stability.

## Conclusion

With further development to demonstrate additional measurement properties, the SAFE may prove a valuable tool for clinicians involved in managing the foot with RA, enhancing patient centred care and facilitating communication between clinicians and patients. The SAFE may also have application as an outcome measure for research and evidence-based practice.

